# What Deters Crime? Comparing the Effectiveness of Legal, Social, and Internal Sanctions Across Countries

**DOI:** 10.3389/fpsyg.2016.00085

**Published:** 2016-02-08

**Authors:** Heather Mann, Ximena Garcia-Rada, Lars Hornuf, Juan Tafurt

**Affiliations:** ^1^Department of Psychology and Neuroscience, Duke University, DurhamNC, USA; ^2^Social Science Research Institute, Duke University, DurhamNC, USA; ^3^Department of Economics and IAAEU, University of TrierTrier, Germany; ^4^AG3 ConsultoresBogota, Colombia

**Keywords:** dishonesty, crime, cheating, cross-cultural, deterrence theory, deterrence

## Abstract

The question of what deters crime is of both theoretical and practical interest. The present paper focuses on what factors deter minor, non-violent crimes, i.e., dishonest actions that violate the law. Much research has been devoted to testing the effectiveness of legal sanctions on crime, while newer models also include social sanctions (judgment of friends or family) and internal sanctions (feelings of guilt). Existing research suggests that both internal sanctions and, to a lesser extent, legal sanctions deter crime, but it is unclear whether this pattern is unique to Western countries or robust across cultures. We administered a survey study to participants in China, Colombia, Germany, Portugal, and USA, five countries from distinct cultural regions of the world. Participants were asked to report the likelihood of engaging in seven dishonest and illegal actions, and were asked to indicate the probability and severity of consequences for legal, friend, family, and internal sanctions. Results indicated that across countries, internal sanctions had the strongest deterrent effects on crime. The deterrent effects of legal sanctions were weaker and varied across countries. Furthermore, the deterrent effects of legal sanctions were strongest when internal sanctions were lax. Unexpectedly, social sanctions were positively related to likelihood of engaging in crime. Taken together, these results suggest that the relative strengths of legal and internal sanctions are robust across cultures and dishonest actions.

## Introduction

The question of what deters crime is of interest to social science researchers and policy-makers alike. Are decisions to engage in crime influenced by the threat of legal consequences? Are they influenced by threats of judgment from friends or family? Are they influenced by the potential for internal feelings of guilt? These questions are relevant to any society, as dishonesty can be extremely costly. For example, it is estimated that for most countries, losses due to tax evasion are greater than the total amount spent on healthcare (The Tax Justice Network, 2011).

In this paper, we compare the relative impacts of legal, friend, family, and internal sanctions on minor, non-violent crimes. We refer to these transgressions as dishonest because they benefit the individual at society’s expense. We define dishonest actions as those that violate a formal or informal social rule for personal gain; by this definition, lying, cheating, and stealing may all be considered facets of dishonesty. It is worth noting that some dishonest actions harm other individuals rather than society at large; for example, lying to one’s partner likely violates the (spoken or unspoken) relationship contract, but does not directly harm society. Typically, dishonest actions that harm the collective (e.g., underreporting income on one’s taxes) are also subject to legal penalties; the present research focuses on violations of this nature.

While much of the existing research focuses on a single category of sanctions on crime, in the present study, we compare the relative impacts of legal, friend, family, and internal sanctions and consider their interactions. By drawing on a participant sample from five countries in distinct cultural regions, we examine whether the deterrent effects of legal, social, and internal sanctions are consistent across individuals from different cultural backgrounds.

### What Deters Crime?

A sizeable body of research on the subject of what deters crime has focused on the effectiveness of legal sanctions. This research stems from deterrence theory, which posits that legal sanctions deter citizens from engaging in criminal activity. This theory, grounded in the rational actor approach, is based on the notion that people choose whether or not to commit a crime by weighing the potential benefits of getting away with it against the potential consequences of getting caught (Becker, 1968). Consequences are considered in terms of both severity of the punishment and the probability of being caught. Building on thinking of 18th century philosophers Beccaria (1963 [1764]) and Bentham (1988 [1789]), and revived in the 1960s, deterrence theory has generated much research and heated debate, with some researchers arguing that legal sanctions have no effect at all (e.g., Fattah, 1983).

Recently, Rupp (2008) conducted an impressive meta-analysis synthesizing the findings from 700 studies testing the deterrence hypothesis, spanning economics, sociology, psychology, and criminology. Detailed information about each study, including aspects of study design (cross-sectional, experimental, survey, etc.), participant sample, categories of sanctions measured, and information about the authors and journal were coded and analyzed. On the whole, this meta-analysis favored rejecting the null hypothesis that legal sanctions have no deterrent effect on crime. Furthermore, the probability of legal sanctions was found to have a greater deterrent effect than the severity of legal sanctions. In Rupp’s analysis, there was also a clear pattern for legal sanctions to have stronger deterrent effects for minor, non-violent crimes (including tax evasion, speeding, and fraud) than for violent or more serious crimes (including hard drug dealing, sexual assault, and manslaughter). This pattern suggests a categorical difference in the factors deterring minor and more serious crimes. In the present paper, our research scope is limited to the factors influencing minor, non-violent crimes.

A chief criticism of deterrence theory has been its neglect of non-economic factors that may influence crime (Meier et al., 1984; Williams and Hawkins, 1986). Researchers from sociology and other traditions have suggested that non-economic sanctions have at least as much potential to impact criminal behavior (Wrong, 1961; Grasmick and Green, 1980; Mazar et al., 2008). One type of non-economic sanction considered is judgment by friends and family, which some have referred to as the threat of social embarrassment (Grasmick and Bursik, 1990; Cochran et al., 1999). Research from psychology and sociology suggests that people are highly sensitive to social evaluation (Dickerson et al., 2008). However, according to Rupp’s meta-analysis, of the 2534 variables examined in survey studies, only 6.2% assessed the perceived probability of punishment by friends or family, 4.1% assessed the perceived severity of punishment by friends or family, and 2.8% assessed the perceived probability of detection by friends, family or others. Results from the meta-analysis indicated that the probability of punishment by friends or family was at least as strong a deterrent as the probability of legal punishment, and the severity of punishment by friends or family, though less powerful than the probability effects, was at least as strong a deterrent as the severity of legal punishment.

Finally, there appears to be increasing awareness that in addition to external sanctions, internal sanctions such as feelings of guilt may be important deterrents of crime. Though focused on dishonest rule violations rather than illegal actions *per se*, Mazar et al. (2008) posited that dishonesty is regulated largely by the internal desire to maintain a positive self-concept, which is weighted against the potential material benefits of breaking the rules. In support of this theory, experiments showed that increasing the flexibility with which people can categorize their dishonest actions (e.g., cheating for tokens with monetary value rather than money itself) encourages dishonesty, and conversely, that drawing attention to moral standards mitigates dishonesty. Furthermore, several experimental studies have found that increasing financial incentives for behaving dishonestly has surprisingly little impact on dishonest behavior (Wiltermuth, 2011; Gino et al., 2012; John et al., 2014; Weisel and Shalvi, 2015). For example, John et al. (2014) found that participants were just as likely to cheat on a trivia game when they were paid 5 cents per self-reported correct answer as when they were paid 25 cents per self-reported correct answer.

### Considering Interactions Between Sanctions

An additional question sometimes raised by researchers is whether the deterrent effects of legal, social, and internal sanctions are independent of one another. Some scholars have raised the interesting hypothesis that the deterrent effects of legal sanctions should be most evident when moral commitments (i.e., internal sanctions) are weak (Zimring, 1971; Silberman, 1976). Evidence supporting this interaction hypothesis was reported by Silberman (1976), and more recently by Wenzel (2004), who found that in a sample of Australian citizens, penalties for tax evasion had a deterrent effect only when internal sanctions were lax. However, Grasmick and Green (1980, 1981) argued against this interaction hypothesis in favor of additive effects.

### An Integrated Deterrence Framework

While many researchers who have explored the impacts of social and internal sanctions on crime have contrasted their approaches with deterrence theory, Grasmick and Bursik (1990) proposed that the deterrence framework could be extended to incorporate social and internal sanctions. They designed a survey with questions assessing the perceived probability and severity of legal, social, and internal sanctions. Sanction threat variables, computed as the product of perceived probability and severity, were entered as predictors in regression models for three illegal actions: tax evasion, theft and drunk driving. Across the three actions, both legal sanctions and internal sanctions were significant deterrents, but internal sanctions had the stronger deterrent effect. Surprisingly, the deterrent effect of social sanctions was not significant.

### Are People Deterred From Crime the Same Way Everywhere?

The limited number of studies employing Grasmick and Bursick’s extended deterrence framework support their original findings that legal and internal sanctions deter crime, with internal sanctions having the stronger deterrent effect (Grasmick et al., 1993a,b; Cochran et al., 1999; Kobayashi et al., 2001). Notably, these studies have failed to provide evidence for a deterrent effect of social sanctions; the reason these effects differ from those reported in Rupp’s meta-analysis is not entirely clear. Moreover, these studies have been conducted on Americans, raising the question of whether the findings are robust across cultures. (Kobayashi and colleagues’ study is an exception, including both Americans and Japanese, but the researchers do not compare the strengths of deterrent effects across cultures. Wenzel (2004) also reports similar effects in an Australian sample.)

In his meta-analysis of the deterrence literature, Rupp found that the deterrent effect of legal sanctions varied according to the country under study. For example, support for the deterrence hypothesis was stronger in studies conducted in Germany and the UK than in studies conducted in Canada (Rupp, 2008). However, comparisons in Rupp’s analysis were limited to select Western nations with sufficient numbers of studies testing the effects of legal deterrents. Furthermore, the deterrence effect was also found to vary according to authors’ home country and country of publication, raising the possibility that the cross-country variation observed was related to author biases. Comparing culturally distinct countries within a single study overrides these issues, and allows for a more rigorous assessment of whether the relative effects of legal, social, and internal sanctions are consistent across cultures.

### The Present Research

Building on the extended deterrence framework of Grasmick and Bursik (1990), we compared the deterrent effects of legal, social, and internal sanctions on minor, non-violent crimes within a single study. To compare the relative influences of these deterrents across cultures, we administered our study to an international participant sample from five countries: China, Colombia, Germany, Portugal, and USA. These countries are based in distinct cultural regions of the world, namely Confucian (China), Catholic Latin America (Colombia), Protestant Europe (Germany), Catholic Europe (Portugal), and English-speaking (USA), according to cultural mapping by Inglehart and Welzel (2010). The countries sampled differ along two broad cultural dimensions identified by Inglehart and Baker (2000) and Inglehart and Welzel (2010): traditional vs. secular-rational values and survival vs. self-expression values. Within each country, we administered a survey to two participant groups: students at public universities, and the general public at coffee shops in major cities.

We designed a survey with four sanction categories: legal, friends, family, and internal. While the threats of judgment from friends and family have traditionally been grouped together as social sanctions, we considered that judgment from friends and judgment from family might have different motivational impacts, which might vary across cultures. For example, the threat of family sanctions, but not friend sanctions, may be stronger in more traditional cultures. The first three sanction categories (legal, friends, and family) focus on negative consequences that are *external* to the individual. The final category focuses on internal consequences, namely on feelings of guilt. Other researchers used the term shame rather than guilt in referring to internal sanctions (Grasmick et al., 1993a; Kobayashi et al., 2001). In the psychological literature, guilt is typically construed as feeling badly over one’s actions, while shame is typically construed as feeling badly over who one is (Tangney, 1998). Because guilt is triggered by violating internal moral standards, and may or may not induce shame, our internal sanctions measure asks about feelings of guilt rather than shame.

Participants were first asked to report the likelihood of engaging in seven minor, non-violent crimes, including parking illegally, bribing a police officer, and tax evasion. For each action, participants were asked to rate both the probability of detection and severity of punishment across each of the four sanction categories.

Our primary research questions were whether legal, social, and/or internal sanctions negatively predict the likelihood of engaging in dishonesty, and whether deterrent effects are consistent across cultures. Based on previous research suggesting the primacy of internal influences, we hypothesized that internal sanctions would have the strongest deterrent effect across cultures. In addition, we tested the interaction hypothesis that the effects of legal sanctions are stronger when internal sanctions are lax.

## Materials and Methods

This study was administered with approval from Duke University’s Institutional Review Board for Non-Medical Research. All participants provided their informed written consent.

### Participants

A total of 1,251 individuals completed the crime sanctions survey. To ensure that our participant sample reflected the cultures of our countries of interest, we limited our analyses to those who were native residents of each country (born in and currently residing in the country). In addition, twelve individuals were excluded due to technical issues or internal reasons, leaving 1,100 participants in our final sample. Approximately half of the participants (*N* = 586) were students recruited from public universities, while the other half (*N* = 514) were members of the general public, recruited in coffee shops from the same cities. Participants were sampled from five countries: China, Colombia, Germany, Portugal, and USA.

### Crime Sanctions Survey

All survey materials were translated into the native language of participants from each country, using a forward–backward translation procedure. Participants completed the survey individually on iPads. An instructions screen informed them that they would be asked different questions about the same actions, and that they should respond as honestly as possible. They were assured that their responses were confidential and anonymous. All participants were first asked about the likelihood that they would engage in seven minor, non-violent crimes, in the form, “How likely are you to ____?” Participants responded on continuous sliding scales ranging from 0 (“not at all likely”) to 10 (“very likely”).

Participants indicated how likely they would be to engage in the following actions:

(1) Omit information on your tax filings in order to pay less income tax
(2) Speed by 15% over the speed limit while driving
(3) Run a red light when nobody is around
(4) Park your car in a no parking zone
(5) Bribe a police officer to avoid getting a speeding ticket
(6) Apply for a government tax credit knowing you are not eligible for it
(7) Fake a signature of a doctor on a government document in order to get an expensive medication for free.

These questions were presented on the same screen in randomized order.

Next, participants were asked to report their perceptions of legal, social, and internal sanctions for each of the seven actions. Participants were asked about two categories of social sanctions, friends and family, resulting in four sanction categories. For each category, participants were asked about the perceived probability of being penalized for engaging in the actions with the following questions:

**Legal probability:** How likely would you be to get caught by the government authorities or police if you…

**Social probability (friends):** How likely would your friends be to find out if you…

**Social probability (family):** How likely would your family be to find out if you…

**Internal probability:** How likely would you be to feel guilty if you…

Continuous sliding scales ranged from 0 (“extremely unlikely” to 10 “extremely likely”). Furthermore, participants were asked to rate the expected severity of the legal, social, and internal consequences, as follows:

**Legal severity:** How bad would the legal penalty be if you…

**Social severity (friends):** How badly would your friends judge you if you…

**Social severity (family):** How badly would your family judge you if you…

**Internal severity:** How badly would you feel if you…

Continuous sliding scales ranged from 0 (“not bad(ly) at all”) to 10 (“extremely bad(ly)”).

The eight question categories were presented in random order, with the seven individual actions presented in random order within each block. In total, participants responded to 56 specific questions about legal, social and internal sanctions.

### Procedure

Students at universities were recruited with flyers and posters advertising a decision-making study where they could earn between $4 and $10. At universities, the study was run in a testing room with 5–8 separate stations for participants. In coffee shops, participants were approached individually by an experimenter, who asked whether they would be interested in participating in a decision-making study with the opportunity to earn between $4 and $10. Coffee shop patrons who agreed to participate completed the survey individually from where they were seated.

Participants first completed a behavioral task on iPads, which involved rolling a virtual die twenty times (adapted from Jiang, 2013; see Mann et al., under review for further detail). Before each roll, participants were instructed to select a side of the die, either top or bottom. They were instructed to remember their chosen side, but were not asked to report choosing top or bottom until they had viewed the outcome of the roll (the screen displayed the number of dots on both top and bottom of the die). Participants were paid the equivalent of ten cents in USD for every dot on the chosen side (the amount and currency were adjusted for each country using the Purchasing Power Parity Index). Therefore, if a participant mentally selected “top” before rolling the die, and the outcome displayed one dot on the top side and six dots on the bottom side, the participant would face a choice as to whether to honestly report having chosen “top,” or whether to dishonestly report having chosen “bottom”. Once the participant indicated their choice, the earnings for that roll were automatically added to their total earnings, displayed at the top of the screen. With this paradigm it is impossible to know for certain whether cheating occurred on any given roll or for any given person. However, in large samples, if cheating did occur, choosing the favorable earnings side (i.e., the side with more dots) on a greater proportion of trials should be correlated with dishonesty.

When participants completed the die task, the experimenter returned and set up the crime sanctions survey on the iPad. This experimenter, who spoke participants’ native language, set up the survey and instructed them to raise their hands should they have any questions. Participants indicated their responses to each question by moving bars along slider scales with their fingers. At the end of the survey, they raised their hand to indicate that they had finished. The experimenter then thanked them for participating and directed them to a payments table (for students) or paid them directly (for general public).

## Results

### Correlations Between Likelihood of Engaging in Crime (Self-Reported) and Observed Dishonest Behavior

We first examined whether self-reported crime was related to dishonesty on the behavioral die task, in which participants could earn more money by cheating. Detailed behavioral results from the die task are reported in Mann et al. (under review); in the present paper, we present only the correlations between our behavioral measure of dishonesty and our self-report data from the crime sanctions survey. Our behavioral measure of dishonesty was the proportion of trials on the die task in which participants reported choosing the side of the die with favorable earnings. Overall, this proportion ranged from 0.56 (Portugal) to 0.60 (USA) indicating a limited but significant level of cheating in every country. We conducted a Pearson correlation between this outcome and self-reported likelihood of engaging in crime, averaged across the seven illegal actions. Across the full sample, this analysis revealed a modest but significant positive correlation (*r* = 0.08, *p* = 0.012).

Examining the correlations for each country separately revealed positive and significant correlation coefficients for Germany (*r* = 0.20, *p* = 0.004) and the USA (*r* = 0.26, *p* < 0.001), while the correlation coefficients for China, Colombia, and Portugal were not significant. Further examination indicated that these results were driven by the student samples in Germany and the USA.

### Comparing Likelihood of Engaging in Crime Across Countries and Cohorts

**FIGURE 1:**
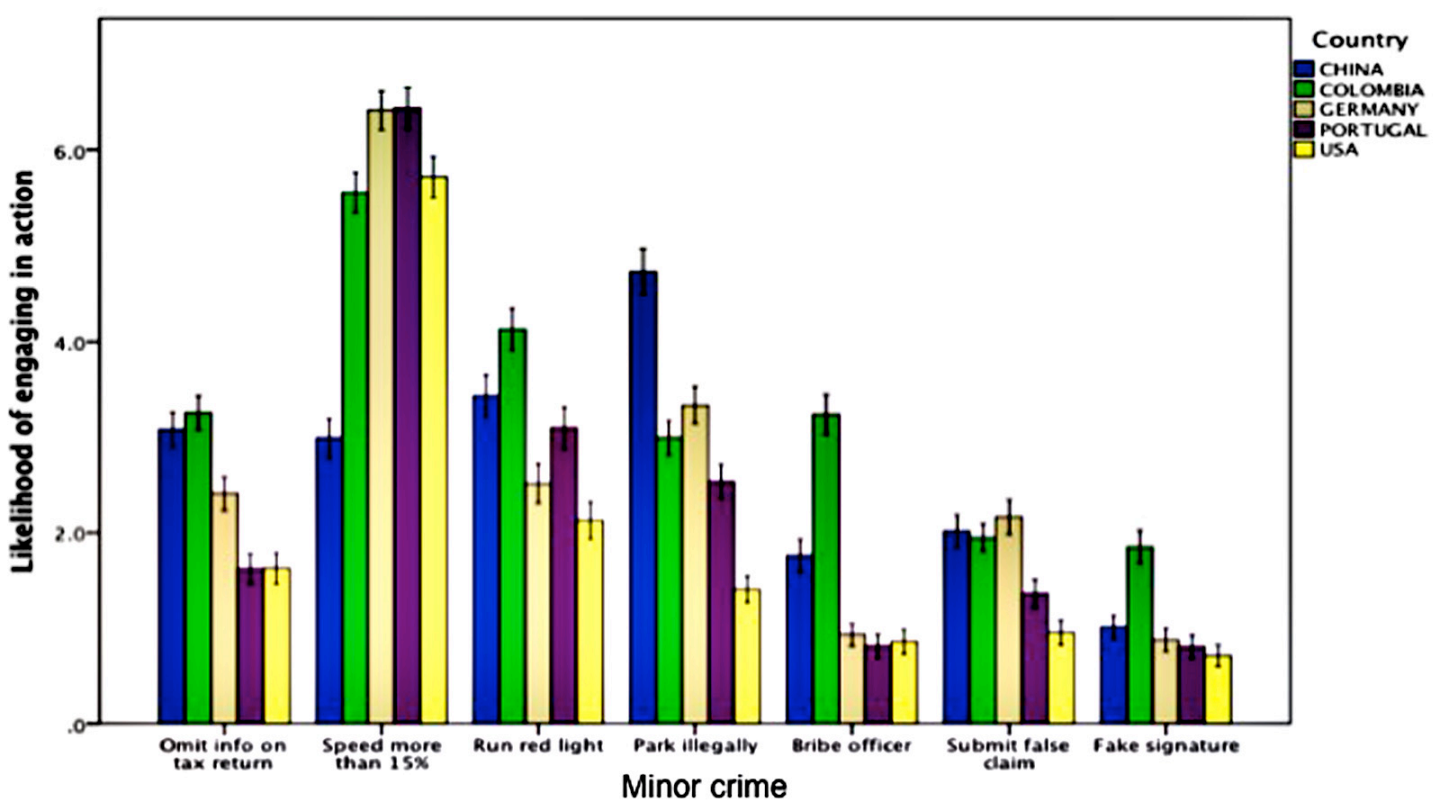
**Self-reported likelihood of engaging in seven specific dishonest actions across countries.** Error bars represent ±1 SEM.

The remaining analyses focus on our self-report data from the crime sanctions survey. We next examined whether likelihood of engaging in crime differed across countries, and across subject groups (students vs. public) within countries. **Table 1** presents the results of separate 5(Country) × 2(Cohort: student vs. public) between-subject ANOVAs conducted on each of the seven scenarios. For every scenario, reported likelihood of engaging in crime differed between countries, and results were significant at a Bonferroni-corrected probability threshold of *p* = 0.007. On the other hand, differences between cohorts were significant for only two scenarios, running a red light and falsely applying for a government tax credit, at a liberal threshold of *p* = 0.05, and for only the latter scenario at the Bonferroni-corrected threshold. Finally, the Country-by-Cohort interaction term was significant for two scenarios (speeding by 15% over the limit and running a red light), but these did not survive the Bonferroni-corrected significance threshold. Based on the limited differences observed between cohorts, along with non-significant effects for cohort in regression analyses, we combine student and public cohorts together in the analyses reported from here on. **Figure 1** shows the reported likelihood of engaging in crime for each scenario across the five countries, illustrating cultural differences.

**Table 1:** Summary of univariate ANOVAs comparing responses across countries and cohorts regarding the likelihood of engaging in seven dishonest actions.

	Statistic	Country	Cohort	Country^∗^Cohort
Omit information on your tax filings in order to pay less income tax	*F*	19.601^∗∗∗^	0.013	0.655
	*$\eta_{p}^{2}$*	0.068	0.000	0.002
Speed by 15% over the speed limit while driving	*F*	44.898^∗∗∗^	0.772	2.374^∗^
	*$\eta_{p}^{2}$*	0.145	0.001	0.009
Run a red light when nobody is around	*F*	13.748^∗∗∗^	6.633^∗^	1.818^∗^
	*$\eta_{p}^{2}$*	0.049	0.006	0.007
Park your car in a no parking zone	*F*	39.500^∗∗∗^	0.706	0.910
	*$\eta_{p}^{2}$*	0.129	0.001	0.003
Bribe a police officer to avoid getting a speeding ticket	*F*	43.289^∗∗∗^	2.551	0.529
	*$\eta_{p}^{2}$*	0.139	0.002	0.002
Apply for a government tax credit knowing you are not eligible for it	*F*	10.879^∗∗∗^	17.983^∗∗∗^	0.982
	*$\eta_{p}^{2}$*	0.039	0.017	0.004
Fake a signature of a doctor on a government document in order to get an expensive medication for free	*F*	12.130^∗∗∗^	3.547	0.372
	*$\eta_{p}^{2}$*	0.043	0.003	0.001


*All country differences were significant at a Bonferroni-corrected threshold of *p* = 0.007. At this threshold, no cohort differences were significant except applying for a tax credit knowing you are not eligible, nor were any country by cohort interactions.*

*^∗^*p* ≤ 0.05, ^∗∗^*p* < 0.01, ^∗∗∗^*p* < 0.001.*

### Deterrent Effects of Legal, Social, and Internal Sanctions

We computed legal, friend, family, and internal sanction variables by multiplying the probability and severity ratings for each action in each of the four categories. Sanction threats are commonly understood as the interaction between probability and severity of sanctions (Rupp, 2008), which derives from classical utility theory. We qualify this approach by noting that although our variables are continuous, they are not interval or ratio variables. The probability variables do not represent absolute probability scales, but rather, participants’ perceptions of probability. Acknowledging the limitations of multiplying ordinal variables, for ease of interpretation and for comparison with existing theory and research, we followed tradition in multiplying self-reported probability and severity values to compute the sanction threat variables (e.g., Grasmick and Bursik, 1990; Kobayashi et al., 2001; Wenzel, 2004). For the remaining analyses, we structured our data such that each row represented a particular subject’s response to a particular question.

We first examined the relative importance of the four types of sanctions across all subjects by running linear mixed effects analyses with data from all subjects and questions. These analyses were run in R Core Team (2014), using the lme4 package (Bates et al., 2014). *P*-values were computed with the Satterthwaite approximation, using the lmerTest package (Kuznetsova et al., 2014). Models were estimated using a maximum likelihood (ML) approach. To facilitate interpretation of the parameter estimates, all fixed effects variables and the dependent measure were first standardized to have a mean of 0 and standard deviation of 1.

Results from three mixed effect models are reported in **Table 2.** As a baseline, we ran an initial model with demographic variables (gender, age, minority status, relative earnings, religiosity, and mistrust of others) entered as fixed effects, and likelihood of engaging in crime entered as the dependent measure (Model 1). To account for non-independent responses, item, country, and subjects nested within country were entered as random effects variables. This analysis showed significant effects for gender, age, relative earnings, and mistrust in others. Women were less likely to engage in crime than men, although this did not hold up in subsequent models. Older individuals reported being less likely to engage in crime, while those with higher relative earnings reported being more likely to engage in crime overall. This finding aligns with the work by Piff et al. (2012), which suggests that upper class individuals are less ethical than lower class individuals (See also Ariely and Mann, 2013; Trautmann et al., 2013). Finally, as others have found (Uslaner and Badescu, 2004; Neville, 2012), mistrust in others was related to greater likelihood of engaging in crime.

**Table 2:** Results from linear mixed effects models with ML estimation for likelihood of engaging in crime.

	Model 1		Model 2		Model 3	
			
Fixed effects	*b*	*p*	*b*	*p*	*b*	*p*
(Intercept)	0.005	0.979	0.005	0.000^∗∗∗^	-0.042	0.000^∗∗∗^
FEMALE	-0.037	0.029^∗^	0.010	0.525	0.011	0.495
Age	-0.084	0.000^∗∗∗^	-0.036	0.027^∗^	-0.037	0.024^∗^
MINORITY	-0.012	0.495	-0.017	0.283	-0.020	0.223
Relative Earnings	0.057	0.001^∗∗∗^	0.053	0.001^∗∗∗^	0.051	0.001^∗∗^
Religiosity	-0.006	0.728	0.018	0.285	0.017	0.311
Mistrust	0.043	0.012^∗^	0.033	0.037^∗^	0.031	0.057^†^
LEGAL			-0.091	0.000^∗∗∗^	-0.122	0.000^∗∗∗^
FRIEND			0.026	0.056^†^	0.037	0.029^∗^
FAMILY			0.025	0.080^†^	0.017	0.437
INTERNAL			-0.398	0.000^∗∗∗^	-0.397	0.000^∗∗∗^
LEGAL^∗^FRIENDS					-0.024	0.071^†^
LEGAL^∗^FAMILY					-0.021	0.108
LEGAL^∗^INTERNAL					0.113	0.000^∗∗∗^
FRIEND^∗^FAMILY					-0.009	0.408
FRIEND^∗^INTERNAL					0.007	0.657
FAMILY^∗^INTERNAL					0.023	0.106

**Random effects**	**σ**		**σ**		**σ**	

Subject^∗^Country	0.400		0.371		0.377	
Item	0.460		0.301		0.292	
Country	0.143		0.126		0.133	
Residual	0.776		0.709		0.700	

Log-likelihood	-7836		-6721		-6667	
Likelihood ratio test against previous model			χ_(4)_^2^ = 2231.6	0.000^∗∗∗^	χ_(6)_^2^ = 106.76	0.000^∗∗∗^


*All models include subject, item, and country as random effects variables, with subject nested within country. Fixed effect variables and the outcome variable were standardized for ease of interpretation. Model 1 includes demographic variables of interest as fixed effect terms. Model 2 additionally includes the four sanction variables, resulting in a highly significant model improvement. Model 3 includes two-way interactions terms for the sanction variables, again resulting in highly significant model improvement. From Models 2 and 3, both internal sanctions and legal sanctions show significant deterrent effects on crime, though the effect of internal sanctions is approximately four times greater. Friend and family sanctions are positively related to crime (significantly so for friend sanctions). A highly significant positive interaction between legal and internal sanctions indicates that the deterrent effect of legal sanctions is stronger when internal sanctions are low.*

*^†^*p* < 0.10, ^∗^*p* < 0.05, ^∗∗^*p* < 0.01, ^∗∗∗^*p* < 0.001.*

Model 2 built on Model 1 to examine the effects of external and internal sanction threats. Including legal, friends, family, and internal sanctions as continuous fixed effect variables resulted in a highly significant model improvement over Model 1, according to a log likelihood ratio test (χ_(4)_^2^ = 2231.6, *p* < 0.001). As can be seen from the table, beta values for legal and internal sanctions were negative and highly significant, indicating that the greater the sanction threat, the lower an individual’s reported likelihood of engaging in crime. Although both legal and internal sanctions predicted unique variance in the model, it is also worth noting that the beta value for internal sanctions (*b* = –0.398; *t*(5488) = –27.253) was five times the magnitude of the beta value for legal sanctions (*b* = –0.091, *t*(5599) = –6.575). In contrast, beta values for friends and family sanctions, though modest and only marginally significant, were positive in sign, indicating that greater threats of social judgment, whether from friends or family, predicted *greater* likelihood of engaging in crime. We return to this finding in the Discussion section.

Finally, Model 3 built on Model 2 by including two-way interaction terms for the sanction threats as fixed effect variables (interaction terms were computed from the standardized sanction threat variables). Including interaction terms led to significant model improvement over Model 2 (χ_(6)_^2^ = 106.7, *p* < 0.001). We were interested in testing the interaction hypothesis that when internal sanctions (i.e., feelings of guilt) are weak, legal sanctions have a stronger deterrent effect on crime. In support of this hypothesis, we observed a significant, positive interaction between internal sanctions, and legal sanctions. Similar findings were reported by Silberman (1976), and Wenzel (2004). Grasmick and Green (1980) also reported results that were similar in direction though not significant.

To further explore this effect, we conducted follow-up moderation analyses for each of the seven crimes, using Hayes’ process model which follows Baron and Kenny’s (1986) approach (Hayes, 2013). Internal sanctions moderated the effect of legal sanctions for four of the seven crimes (speeding, running a red light, parking illegally, and bribing an officer). For each of these crimes, the negative effect of legal sanctions was stronger when internal sanctions were weak.

In Model 3, the effect of friend sanction threats was positive and significant, and the effect of family sanction threats positive though not significant. In order to gain insight into the unexpected positive relationship between social sanction threats and likelihood of illegal actions, we conducted an additional linear mixed model analysis with standardized probability and severity sanction variables entered as separate fixed effect variables (**Table 3**). Demographic variables were also included in the model, with item, country, and subjects nested within country again entered as random effects variables. This analysis revealed that the probability variables for both friends and family sanctions, where subjects rated how likely their friends or family would be to find out if they acted illegally, were significant *positive* predictors of illegal actions. The severity of family judgment was a significant deterrent of illegal actions, while the severity of friends’ judgment did not significantly predict illegal action.

**Table 3:** Results from a linear mixed effects models (ML estimation) for likelihood of engaging in crime, with probability and severity ratings for legal, friend, family, and internal sanctions entered as predictors, in addition to demographic variables.

Fixed effects	*b*	*p*
(Intercept)	0.006	0.962
FEMALE	0.014	0.387
Age	-0.020	0.223
MINORITY	-0.017	0.296
Relative Earnings	0.039	0.012^∗^
Religiosity	0.027	0.092^†^
Mistrust	0.029	0.063^†^
Legal (Probability)	-0.038	0.002^∗∗^
Legal (Severity)	-0.071	0.000^∗∗∗^
Friend (Probability)	0.091	0.000^∗∗∗^
Friend (Severity)	0.010	0.527
Family (Probability)	0.093	0.000^∗∗∗^
Family (Severity)	-0.114	0.000^∗∗∗^
Internal (Probability)	-0.123	0.000^∗∗∗^
Internal (Severity)	-0.280	0.000^∗∗∗^

**Random effects**	**σ**	

Subject^∗^Country	0.372	
Item	0.262	
Country	0.141	
Residual	0.676	

Log likelihood	-6474	


*Fixed effects variables and the outcome variable were standardized for ease of interpretation. Subject, item, and country were as random effects variables, with subject nested within country. For legal and internal sanctions, both probability and severity ratings were negatively related to crime, with severity ratings having somewhat stronger effects. For friend and family sanctions, probability of being detected was *positively* related to crime; severity of judgment from family was negatively related to crime, while severity of judgment from friends was not significant.*

*^†^*p* < 0.10, ^∗^*p* < 0.05, ^∗∗^*p* < 0.01, ^∗∗∗^*p* < 0.001.*

Rupp’s meta-analysis and common consensus indicate that the probability of legal sanctions has a stronger deterrent effect than the severity of legal sanctions. In contrast, in our data, sanction severity had a stronger deterrent effect than sanction probability, for both the legal and internal sanction categories.

### Do the Effects of Sanctions Vary Across Countries?

Our next question was whether the deterrent effects of legal, friend, family, and internal sanctions were consistent or variable across countries. **Table 4** presents the results of linear mixed models conducted separately for each country. Standardized demographics, sanction variables, and two-way sanction interaction terms were entered as fixed effect predictors, with subject and item entered as random effects. Notably, the effect of relative earnings on engaging in crime was significant only for China and Colombia, whereas for the American sample, the effect of relative earnings was negative and non-significant. Thus, when examined at the country level, our data diverges from Piff et al. (2012) finding that upper class individuals demonstrated more unethical behavior than lower class individuals in an American sample.

**Table 4:** Results from linear mixed effects models (ML estimation) for likelihood of engaging in crime, conducted separately for each country.

	China		Colombia		Germany		Portugal		USA	
					
Fixed effects	*b*	*p*	*b*	*p*	*b*	*p*	*b*	*p*	*b*	*p*
(Intercept)	0.009	0.921	0.147	0.246	-0.116	0.406	-0.086	0.624	-0.254	0.151
FEMALE	-0.031	0.504	-0.036	0.356	-0.010	0.745	0.023	0.515	0.071	0.025^∗^
Age	-0.173	0.054^†^	-0.067	0.108	-0.036	0.253	-0.072	0.053^†^	-0.013	0.595
MINORITY	-0.111	0.029^∗^	-0.043	0.354	-0.012	0.818	-0.053	0.212	0.023	0.273
Relative Earnings	0.192	0.000^∗∗∗^	0.111	0.005^∗∗^	0.033	0.234	0.042	0.241	-0.036	0.241
Religiosity	-0.033	0.509	0.075	0.044^∗^	0.024	0.463	0.043	0.223	-0.022	0.483
Mistrust	0.017	0.690	0.044	0.207	-0.004	0.909	0.076	0.040^∗^	0.037	0.266
LEGAL	-0.235	0.000^∗∗∗^	-0.019	0.554	-0.116	0.000^∗∗∗^	-0.055	0.053^†^	-0.127	0.000^∗∗∗^
FRIEND	0.102	0.006^∗∗^	0.027	0.422	0.025	0.527	-0.004	0.910	-0.067	0.083^†^
FAMILY	0.077	0.099^†^	0.036	0.269	-0.058	0.083^†^	0.067	0.050	0.033	0.275
INTERNAL	-0.418	0.000^∗∗∗^	-0.457	0.000^∗∗∗^	-0.433	0.000^∗∗∗^	-0.373	0.000^∗∗∗^	-0.244	0.000^∗∗∗^
LEGAL^∗^FRIEND	-0.024	0.461	-0.045	0.109	-0.011	0.720	-0.015	0.587	0.005	0.856
LEGAL^∗^FAMILY	-0.040	0.275	0.006	0.831	-0.023	0.439	-0.028	0.278	-0.068	0.007^∗∗^
LEGAL^∗^INTERNAL	0.179	0.000^∗∗∗^	0.021	0.456	0.143	0.000^∗∗∗^	0.090	0.000^∗∗∗^	0.085	0.000^∗∗∗^
FRIEND^∗^FAMILY	-0.002	0.940	-0.021	0.400	-0.008	0.755	0.000	0.984	0.008	0.674
FRIEND^∗^INTERNAL	-0.019	0.605	0.028	0.365	0.005	0.897	0.013	0.679	0.018	0.558
FAMILY^∗^INTERNAL	-0.005	0.893	-0.014	0.656	0.085	0.006^∗∗^	-0.018	0.541	0.039	0.137

**Random effects**	**σ**		**σ**		**σ**		**σ**		**σ**	

Subject	0.175		0.404		0.283		0.335		0.358	
Item	0.028		0.290		0.334		0.432		0.408	
Residual	0.509		0.730		0.654		0.652		0.595	


*The outcome variable was standardized, and standardized demographics, legal, friend, family, and internal sanctions, and two-way sanction interaction terms were entered as fixed effect variables. Subject and item were entered as random effect variables.*

*^†^*p* < 0.10, ^∗^*p* < 0.05, ^∗∗^*p* < 0.01, ^∗∗∗^*p* < 0.001.*

As can be seen from the table, the deterrent effect of internal sanctions was highly significant across all five countries. The deterrent effect of legal sanctions was significant in China, Germany, and USA, marginally significant in Portugal, and not significant in Colombia. Finally, the positive interaction between legal and internal sanctions was significant in every country except Colombia.

To determine whether the strength of sanction threats varied significantly across countries, we ran a linear mixed effects model with standardized sanction variables and individual countries entered as fixed effect variables, in addition to sanction threat by country interaction terms. Country variables were coded using effect coding instead of dummy coding such that each country’s mean could be compared against the grand mean. As is the case for dummy coding, with effect coding for k groups, only k–1 groups can be estimated according to the degrees of freedom. In order to report parameter estimates for all five countries, we ran the linear mixed effects model twice with a different country excluded from estimation each time, and reported the parameters for all five countries in **Table 5.** Other parameters in the model are not affected by the country that is excluded from effect coding.

**Table 5:** Results from a linear mixed effects model (ML estimation) for likelihood of engaging in crime, with demographics, sanction variables, and countries included as fixed effect variables.

Fixed effects	*b*	*p*
(Intercept)	2.578	0.000^∗∗∗^
FEMALE	0.033	0.499
Age	-0.120	0.016^∗^
MINORITY	-0.047	0.339
Relative Earnings	0.153	0.002^∗∗^
Religiosity	0.051	0.310
Mistrust	0.106	0.029^∗^
LEGAL	-0.329	0.000^∗∗∗^
FRIEND	0.082	0.048^∗^
FAMILY	0.073	0.105
INTERNAL	-1.201	0.000^∗∗∗^
CHINA	0.120	0.282
COLOMBIA	0.614	0.000^∗∗∗^
GERMANY	-0.071	0.461
PORTUGAL	-0.129	0.185
USA	-0.534	0.000^∗∗∗^
LEGAL^∗^CHINA	-0.497	0.000^∗∗∗^
LEGAL^∗^COLOMBIA	0.299	0.000^∗∗∗^
LEGAL^∗^GERMANY	0.004	0.960
LEGAL^∗^ PORTUGAL	0.127	0.107
LEGAL^∗^USA	0.067	0.417
FRIEND^∗^CHINA	0.296	0.000^∗∗∗^
FRIEND^∗^COLOMBIA	-0.015	0.851
FRIEND^∗^GERMANY	-0.035	0.706
FRIEND^∗^PORTUGAL	-0.049	0.560
FRIEND^∗^USA	-0.198	0.012^∗^
FAMILY^∗^CHINA	0.027	0.791
FAMILY^∗^COLOMBIA	0.014	0.859
FAMILY^∗^GERMANY	-0.111	0.216
FAMILY^∗^PORTUGAL	0.026	0.760
FAMILY^∗^USA	0.043	0.604
INTERNAL^∗^CHINA	0.083	0.414
INTERNAL^∗^COLOMBIA	-0.153	0.055^†^
INTERNAL^∗^GERMANY	-0.213	0.007^∗∗^
INTERNAL^∗^PORTUGAL	-0.131	0.107
INTERNAL^∗^USA	0.414	0.000^∗∗∗^

**Random effects**	**σ**	

Subject	1.123	
Item	0.915	
Residual	2.148	
Log likelihood	-13158	


*Demographic variables, sanction variables, and the outcome variable were standardized for ease of interpretation. Two-way interaction terms between sanction and country variables were also included in the model. Subject and item were entered as random effect variables. Effects coding was used for countries, such that the reported parameter estimates compare each country’s mean against the grand mean. The analysis was run twice with a different country excluded in the deviation time each time, so that parameter estimates for all five countries could be reported.*

*^†^*p* < 0.10, ^∗^*p* < 0.05, ^∗∗^*p* < 0.01, ^∗∗∗^*p* < 0.001.*

As can be seen from **Table 5**, country main effects were significant only for Colombia and USA; overall, Colombians reported greater-than-average likelihood of engaging in illegal actions (*b* = 0.614, *p* < 0.001), while Americans reported less-than-average likelihood. Sanction by country interactions terms allowed us to address the question of whether the deterrent effects of sanctions varied according to country. Legal sanctions were found to have stronger deterrent effects for China and weaker deterrent effects in Colombia. The reverse deterrent effect of friend sanctions was particularly strong in China relative to the other countries (positive interaction term) whereas a negative interaction term was observed for USA. With regard to family sanctions, no significant differences were observed across countries. Finally, internal sanctions had significantly stronger deterrent effects in Germany, and marginally stronger deterrent effects in Colombia, whereas in the USA, internal sanctions were weaker relative to other countries.

### Do Deterrent Effects Vary Across Actions?

Until this point, variation in specific crimes was treated as a nuisance variable. To compare the deterrent effects of sanction threats across the seven actions, we conducted separate linear regression analyses for each action. Legal, friend, family, and internal sanctions for the specific crime were entered as predictors, along with demographic variables (predictor variables were unstandardized, as the standardized beta values are computed for these models). First, the series of linear regression analyses was run on the full sample, not distinguishing subjects based on country. These analyses were then repeated on subjects from each of the five countries separately.

**FIGURE 2:**
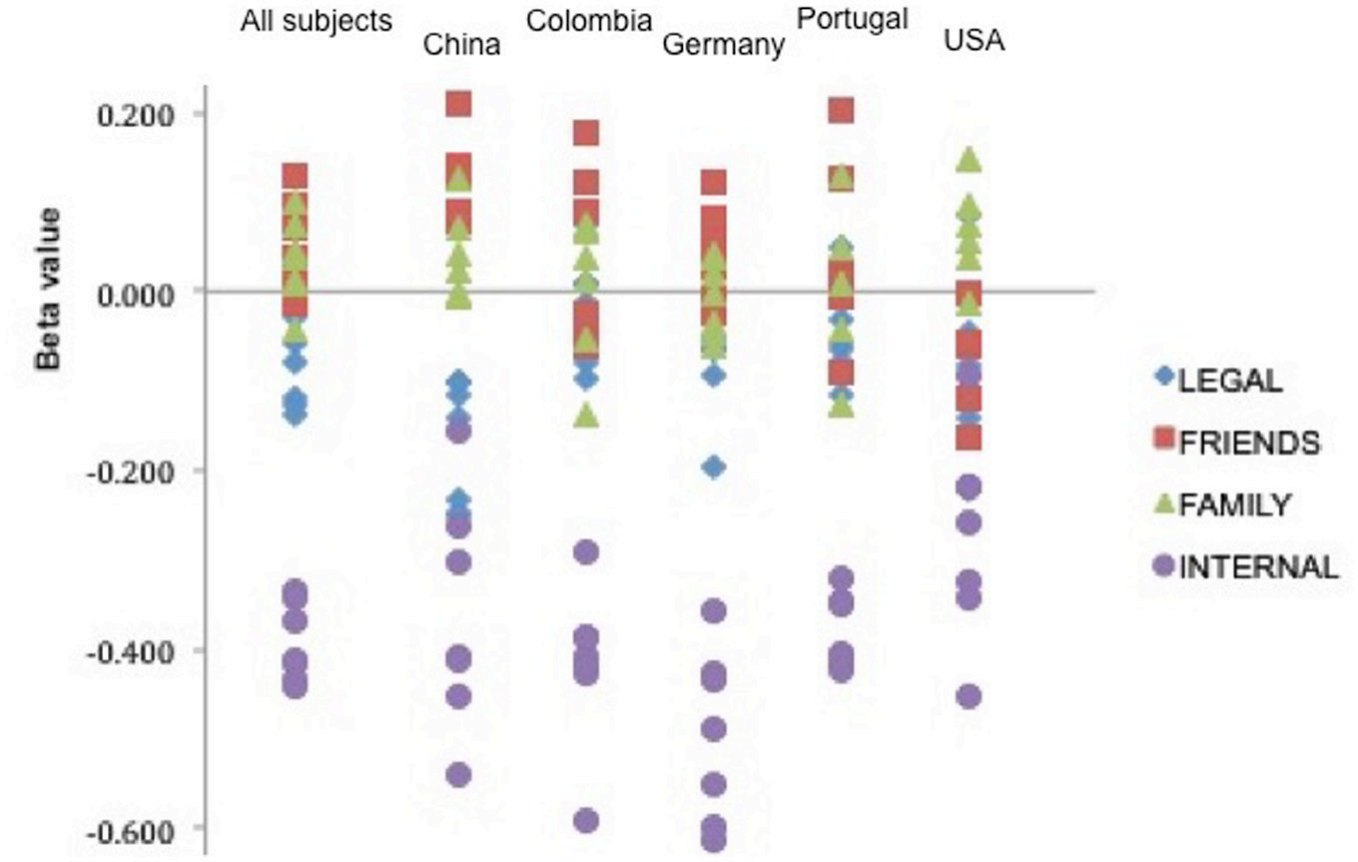
**Summary of the beta values for legal, friends, family, and internal product variables entered as predictor variables in linear regression analyses.** For each item, sanction product variables were entered as predictors with self-reported likelihood of engaging in the action entered as the dependent measure.

The beta values for legal, friend, family and internal sanction threats for each series of regression analyses are depicted in **Figure 2.** As can be seen from the figure, with limited exceptions, the deterrent effects of internal sanction threats are non-overlapping with the deterrent effects of the other categories of sanction threats.

## Discussion

Building on a substantial literature examining the deterrence hypothesis, the present research compared the effectiveness of legal, social (both friend and family), and internal sanctions on deterring minor, non-violent crimes in an international sample spanning five countries. Replicating the findings of others (Grasmick and Bursik, 1990; Grasmick et al., 1993a,b; Cochran et al., 1999; Kobayashi et al., 2001; Wenzel, 2004), we found internal sanctions to have the strongest deterrent effect on crime. This pattern was observed in every country studied, indicating that the primacy of internal sanctions is robust across cultures. In line with deterrence research, legal sanctions were also found to have a significant though weaker overall effect. The effect of legal sanctions was significant in China, Germany, and USA, marginally significant in Portugal, and non-significant in Colombia, suggesting variability across cultures in the extent to which legal sanctions effectively deter crime. The relative effects of internal and legal deterrents were also robust across actions, with internal sanctions usurping legal sanctions for every action in every country, with only one exception (bribing a police officer by Americans).

Some researchers have proposed that the deterrent effects of legal sanctions are stronger when internal sanctions are lax, though others have argued in favor of purely additive effects (Grasmick and Green, 1980, 1981). Supporting the interaction hypothesis, we observed a significant positive interaction between legal and internal sanction threats, an effect also observed by Wenzel (2004) in his study of tax evasion among Australian citizens. In our international sample, the interaction was evident in every country except Colombia. Follow-up moderation analyses showed that the effect of legal sanctions was significant only when internal sanctions were lax; however, the moderation was significant for only four of the seven illegal actions (in contrast to Wenzel’s findings, the effect was not significant for tax evasion). These results suggest that the interaction between legal and internal sanctions may depend on the particular action.

### Social Influences on Crime

An unexpected finding was the positive relationship observed between social sanctions and crime. Overall, the effect of friend sanctions was positive and significant. Examining countries separately, a significant or marginally significant positive effect for either friend or family sanctions was observed in every country except Colombia. To better understand these effects, we conducted additional analyses with probability and severity sanction variables entered as separate predictors. In every country, probability of being found out by friends was positively related to likelihood of acting illegally; the same was true for probability of being found out by family in every country except Germany. Although this result was not anticipated, we speculate that both probability of engaging in crime and probability of being found out by friends and family may be related to a third underlying variable, namely the extent to which the action is normative. For example, if bribing a police officer is a widely practiced behavior in a particular society, an individual in that society may be more likely to practice the behavior, and her friends may be more likely to know about it, than an individual in a society where bribing police is not normative. In line with the hypothesis that social norms influence dishonesty, Gino et al. (2009) found that individuals were more likely to cheat on a test after observing an in-group member cheat, while observing an out-group member cheat had the opposite influence on dishonest behavior.

Another possibility is that people who engage in crime give more thought to the possibility of others finding out about their actions. For example, if a person regularly parks illegally, she may be more likely to think about (and overestimate) the possibility of being found out by friends relative to others who have rarely contemplated this crime. Thus, normativity and degree of cognitive reflection are two potential explanations for the observed positive relationship between probability of being found out and probability of engaging in crime. Since we cannot test third variable explanations with the given data, we recommend that future research examining the relationship between social sanctions and dishonest behavior incorporate these variables.

We are not aware of any other study reporting a positive relationship between social sanction threats and likelihood of engaging in crime. Grasmick and Scott (1982) observed deterrent effect of social sanctions on crime, while several other studies comparing legal, social, and internal sanctions have failed to find deterrent effects of social sanctions (Grasmick and Bursik, 1990; Grasmick et al., 1993a; Cochran et al., 1999; Kobayashi et al., 2001). Taken together, what can we make of these results? Do they imply that threat of social judgment does not impact likelihood of engaging in crime? Such a conclusion seems highly unlikely in light of a vast body of research illustrating the power of social norms (Cialdini and Goldstein, 2004; Fehr and Fischbacher, 2004). We propose instead that the power of social norms occurs primarily through their internalization as moral standards by members of society (Campbell, 1964). When individuals identify with their society, they adopt society’s moral standards as personal moral standards (Wenzel, 2004). The threat of personal judgment (feelings of guilt) for one’s own transgressions then becomes a more effective deterrent than the judgment of friends or family. In support of this theory, Wenzel (2004) found in an Australian sample that social norms had a significant effect on tax evasion only for those who did not identify as Australian (i.e., those who presumably did not internalize the prevailing standards).

Some scholars have proposed that legal sanctions deter crime not through material disincentives but by increasing the level of social condemnation that results from a dishonest action (Tittle and Logan, 1973; Williams and Hawkins, 1986). According to this theory, if a person acts dishonestly, other people will judge her more harshly for her action if it is against the law, and it is this increased threat of social judgment that accounts for the legal deterrent effect. Interestingly, we observed a marginally significant negative interaction between legal and social sanctions, implying that the legal deterrents were more effective when social sanctions were stronger. In his study of tax evasion, Wenzel (2004) observed a similar effect (though it was only evident for those who did not identify as Australian citizens). These results provide tentative evidence for synergistic effects when legal and social sanctions operate in tandem.

### Implications

Kobayashi et al. (2001) examined differences in workplace compliance between Japanese and American employees, and found that these differences could be accounted for by differences in perceived internal, social and management (regulatory) sanctions. In contrast, while we observed country differences in terms of likelihood of engaging in specific crimes, these cross-cultural differences in likelihood of engaging in crime were not entirely accounted for by differences in sanctions. For six of the seven actions in our study, differences in legal sanctions across countries explained some of the variation in country-level differences in crime, while differences in social and internal sanctions were unrelated to country variation. These results raise the interesting possibility that cultural drivers of dishonesty are not entirely captured by sanctions. For example, it is possible that cultural differences in internal or external reward associated with dishonesty account for variation in crime. Further research is needed to understand whether cross-cultural variation in crime is best accounted for by differences in sanctions or differences in other variables.

From a policy perspective, our findings raise the important question of whether policy efforts can change people’s internal moral commitments to honesty and socially upright behavior. In a longitudinal study on drunk driving, Grasmick et al. (1993a) measured intentions to engage in drunk driving in 1982 and 1990, along with perceived legal, social, and internal sanctions, among residents of Oklahoma City. This 8-year interval was characterized by social efforts aimed at reducing drunk driving (for example, Mothers Against Drunk Driving rose to prominence during this time), as well as harsher legal sentences (Jacobs, 1989; Ross, 1994). The study found that intentions to engage in drunk driving indeed diminished over the 8-year period – but that the reduction was primarily accounted for by the threat of internal sanctions, rather than perceived threats of social or legal sanctions. These results suggest that over time, efforts at changing policy and/or social attitudes may translate into internalized morals.

### Limitations and Future Directions

Our study should be qualified in light of limitations. Our data were collected using survey methodology. Directly asking participants to assess the probability and severity of sanction threats after reporting the perceived likelihood of engaging in minor, non-violent crimes has the advantage of enabling direct comparison of legal, social, and internal sanctions. However, this methodology yields results that are correlational, and based on self-report. It is natural to wonder whether social desirability biases influence reports of dishonest or illegal behavior. Although we cannot rule out this possibility, we note that self-report methodologies are commonly used to assess dishonesty (Grasmick et al., 1993a; DePaulo et al., 1996; Cochran et al., 1999; Kobayashi et al., 2001; Ennis et al., 2008). We do acknowledge the possibility that social desirability bias may vary by country (Bernardi, 2006). However, social desirability bias should not affect our main results provided that it does not differentially impact reports of probability or severity of legal, social, or internal sanction threats.

Future research may provide complementary evidence by comparing the strengths and interplay of legal, social, and internal sanctions using experimental methods. For example, future research might examine the hypothesis that internal sanctions derive from social norms by manipulating whether a particular dishonest action is condemned by in-group or out-group members, and then measuring participants’ (a) likelihood of engaging in the dishonest action themselves, and (b) judgment of others who engage in the dishonest action. Furthermore, researchers may vary the extent to which social condemnation of an action is seen as universal or variable, and then assess participants’ own views of the action. Finally, it would be interesting to test the interaction between legal and internal sanctions experimentally. For example, researchers might examine whether manipulating the perceived probability and severity of legal sanctions for illegal downloading differentially impacts downloading behavior for participants with strong versus weak personal morals against piracy.

In addition, our data highlight the need for further research into how income and social class impact moral behavior. There has been some discussion in the literature concerning whether social status influences unethical behavior. This discussion was spurred by Piff and colleagues’ findings that upper class individuals were more likely to violate the law than lower class individuals, and that being primed with an upper class mindset encourages greater levels of unethical behavior (Piff et al., 2012). These findings were based on data from American samples. In the present study, we observed an overall positive relationship between relative earnings and likelihood of engaging in minor, non-violent crimes. However, when examining our data at the country level, the relationship was significant and positive for China and Colombia only, whereas the correlation for Americans was negative and non-significant. In another study by Trautmann et al. (2013) employing a representative sample of Dutch participants, the authors did not observe a positive correlation between income and unethical behavior. Trautmann et al. (2013) argued that the relationship between class and unethical behavior is more complex than posited by Piff et al. (2012), a conclusion that appears to be supported by our data (see also Ariely and Mann, 2013). However, we note that both Trautmann et al. (2013) study and the present study provide correlational evidence, whereas Piff et al. (2012) have reported causal evidence in which priming an upper-class mindset leads to more unethical behavior. Further experimental research employing participant samples from different countries is needed to better understand the interesting and potentially complex relationship between income, class, and moral behavior.

## Conclusion

Our findings suggest that across societies and cultures, internalized moral standards exert the most powerful restraints on dishonest behavior (see also Campbell, 1964). Policy efforts aimed at promoting moral internalization may be more effective than efforts aimed at increasing the frequency or probability of legal sentences. However, the process by which internalization occurs remains poorly understood, and marks an important direction for future research aimed at reducing crime and enhancing social welfare.

## Author Contributions

All authors listed, have made substantial, direct, and intellectual contribution to the work, and approved it for publication. All authors were involved in data collection and theoretical framing. Data analysis was conducted by HM.

## Conflict of Interest Statement

The authors declare that the research was conducted in the absence of any commercial or financial relationships that could be construed as a potential conflict of interest.
